# The TT genotype of methylenetetrahydrofolate reductase 677C>T polymorphism increases the susceptibility to pediatric ischemic stroke: meta-analysis of the 822 cases and 1,552 controls

**DOI:** 10.1007/s11033-012-1641-9

**Published:** 2012-05-04

**Authors:** Beata Sarecka-Hujar, Ilona Kopyta, Karolina Pienczk-Reclawowicz, Daniel Reclawowicz, Ewa Emich-Widera, Ewa Pilarska

**Affiliations:** 1 Department of Applied Pharmacy, Medical University of Silesia, Kasztanowa Str 3, 41-200 Sosnowiec, Poland; 2 Department of Neuropediatrics, Medical University of Silesia, Medykow Str 16, 40-752 Katowice, Poland; 3 Department of Developmental Neurology, Medical University of Gdansk, Debinki Str 7, 80-211 Gdansk, Poland; 4 Department of Neurosurgery, Medical University of Gdansk, Debinki Str 7, 80-211 Gdansk, Poland

**Keywords:** *MTHFR* polymorphism, Ischemic stroke, Children

## Abstract

The 677C>T polymorphism within methylenetetrahydrofolate reductase (*MTHFR*) gene is related to an elevated level of homocysteine. Thus it may be considered as a genetic risk factor in ischemic stroke. Apparently studies of this type of polymorphism in childhood stroke have shown conflicting results. We performed meta-analysis of all the data that are available in relation with *MTHFR* polymorphism and the risk of ischemic stroke in children. We searched PubMed (last search dated December 2010) using “*MTHFR* polymorphism”, “ischemic stroke” “child”, “children”, “pediatric stroke” as keywords and reference lists of studies and reviews on the topic. Finally, 15 case–control studies corresponded to the inclusion criteria for meta-analysis. These studies involved the total number of 822 children and adolescents after ischemic stroke and 1,552 control subjects. Fixed or random effects models were used depending on the heterogeneity between the studies. The association between ischemic stroke and 677C>T polymorphism within *MTHFR* gene was observed in three of the studies. The pooled analysis showed that TT genotype of *MTHFR* gene is more common in stroke patients than in controls (*p* = 0.0402, odds ratio = 1.57, 95 % confidence interval 1.02–2.41). The Egger’s test did not reveal presence of a publication bias. The results based on a sizeable group of cases and controls have proved that the 677C>T polymorphism in *MTHFR* gene is associated with the development of ischemic stroke in children.

## Introduction

Ischemic stroke is a relatively rare disease in children. The incidence of the disease comes to about 3 children per 100,000 children per year [1, 2]. Data from family studies and twin studies suggest that genetic risk factors play an important role in the pathogenesis of ischemic stroke [3, 4]. Stroke is a multifactorial disease that may result from interactions between many risk factors: genetic and non-genetic, as well as environmental ones. Rates of recurrence, mortality and neurological deficits in pediatric stroke patients are significant and concern more than two thirds of the affected children [5]. Neurological deficits and post-stroke disability place a heavy burden on the societies.

Case–control approaches are currently being widely used to determine risk factors in ischemic stroke in adults and children. The 677C>T polymorphism in methylenetetrahydrofolate reductase (*MTHFR*) gene is one of the most extensively investigated candidate polymorphisms.* MTHFR* catalyses the reduction of 5,10-methylenetetrahydrofolate to 5-methylenetetrahydrofolate, which is the main form of folate in plasma and a carbon donor for the remethylation of homocysteine to methionine [6]. A single base pair (677C>T) transition in the *MTHFR* gene influences enzyme thermolability, its decreased activity and, in turn, the elevated level of homocysteine. Thus the *MTHFR* polymorphism is suggested to be a genetic risk factor in cerebrovascular diseases, including ischemic stroke [7].

As mentioned earlier, studies of this variant in childhood stroke have revealed conflicting results. Some of the reports have showed the relation between *MTHFR* 677C>T polymorphism and stroke in children [8–10], while other studies have not indicated such correlations [11, 12]. This may be due to the heterogenic character of this type of stroke as different groups of patients may have various sets of genetic factors which predispose to the disease. Some alleles of candidate genes may be strongly associated with the disease in one population, whereas in another this correlation may be weak due to the presence of other genetic factors or specific interactions between genetic and non-genetic factors.

Meta-analysis is a method that enables pooling data from smaller inconclusive studies and yields it with a greater statistical power and allows one to quantify genetic risks more precisely.

We performed meta-analysis of all the data that has been published so far in relation to the risk of ischemic stroke in children with the *MTHFR* 677C>T polymorphism. We aimed to describe the association between TT genotype of *MTHFR* gene and the risk of childhood ischemic stroke by meta-analysis.

## Methods

### Data acquisition

In the first place we identified all articles published before December 2010 on the *MTHFR* 677C>T polymorphism and its association with ischemic stroke in pediatric patients. Two independent investigators searched the literature using PubMed (last search December 2010). All the references cited in the found studies were also reviewed in order to find other published articles that had been indexed by PubMed. The language was limited to English. The key words used for this search were: “*MTHFR* polymorphism”, “ischemic stroke”, “child”, “children”, “pediatric stroke”. Abstracts were not included in the search. The inclusion criteria were as follows: (a) a case–control study, (b) study population or at least a subgroup comprising children (between birth and puberty, till 12 ages) and adolescents (between the ages of 13 and 19); in some studies this population included neonates and perinatal stroke patients (c) confirmed ischemic stroke with magnetic resonance imaging (MRI) or computer tomography (CT). A given study was excluded from this meta-analysis when: (a) genotype or allele frequencies were not reported, (b) study design was other than case–control, (c) the associations between *MTHFR* polymorphism and stroke in adults were investigated, (d) outcome definition was other than ischemic stroke. We attempted to contact the authors of the found reports to get additional data. In case of lack of relevant data or response from the author of a particular work, the study was excluded. Unavailability of data or failure to answer (after repeated attempts) resulted in the exclusion of the study. Of the found publications, 15 studies published during the period from 1998 to 2009 satisfy the inclusion criteria [8–22], including our previously published data [8]. Three of the articles found in PubMed were not included in the meta-analysis because of the overlap of the patients group in other publications [23–25]. We also excluded one publication because the age of the analyzed population ranged from 7 to 36 years [26]. No subgroup containing pediatric population was distinguished so we decided not to the analyze entire data. Data from 15 published studies were included in meta-analysis, comprising in total 822 cases and 1,552 controls.

The meta-analysis was performed in accordance with the Meta-analysis Of Observational Studies in Epidemiology (MOOSE) guidelines [27].

### Statistical analyses

The data were analyzed by means of the MIX 1.7 software [28, 29]. To determine the strength between genetic polymorphism and childhood ischemic stroke pooled odds ratio (OR) was calculated together with 95 % confidence intervals (CI). Heterogeneity between the studies was evaluated using the Dersimonian and Laird’s Q test. If the *p* value was less than 0.05, the heterogeneity was considered statistically significant. The I^2^ metric describes the percentage of the observed between-study variability due to heterogeneity rather than sampling error and was used to quantify heterogeneity. The I^2^ values ranged between 0 and 100 %, with higher values indicating a greater degree of heterogeneity. In case of significant heterogeneity observed between the studies, the pooled OR was estimated using a random effects model, otherwise a fixed effects model was used. Publication bias was examined with the Egger’s regression asymmetry test.

## Results

### Characteristics of the included studies

Thirteen of the analyzed studies involved less than one hundred pediatric stroke patients. The largest group was analyzed by Nowak-Göttl et al. [19], the least group of cases was studied by Morita et al. [13]. The highest distributions of the TT genotype in the patients’ groups were observed by Cardo et al. [18]—29 %, Morita et al. [13]—26 % and Nowak-Göttl et al. [19]—24 %. Frequency of the TT homozygotes among controls was the highest in the studies of Rook et al. [11]—19 %, Komitopoulou et al. [17]—17 % and Kenet et al. [10]—15 %. Additionally, in one publication analysis of the families (both of the parents and their affected offspring) was performed except for a case–control study [8].

Characteristics of individuals included to this meta-analysis is shown in Table 1.

**Table 1:** Characteristics of studies included to the meta-analysis

	Cases						Controls						*p* ^a^	OR^a^	(95 % CI)^a^
	*N*	Age	% Male	CC	CT	TT	*N*	Age	% Male	CC	CT	TT			
Zak et al., Poland 2009 [8]	64	8.7 ± 5.7	54.7	25	30	9	59	9.0 ± 5.9	64.4	32	25	2	0.058	1.49	(0.99–2.24)
Biswas et al., India 2009 [9]	58	<15	–	24	32	2	58	<15	–	48	10	0	<0.001	–	
Kenet et al., Israel 2000 [10]	58	7.2 ± 6.5	51.7	50		8	118	9.3 ± 5.9	65.5	100		18	–	1.06	(0.40–2.70)
Rook et al., Canada 2005 [11]	33	4.2	45.5	17	12	4	21	–	–	9	8	4	0.10	–	
Djordjevic et al., Serbia 2009 [12]	26	8.5 ± 5.1	57.7	9	16	1	50	6.5 ± 4.1	52	23	22	5	0.66	0.36	(0.05–3.25)
Morita et al., USA 2009 [13]	15	–	–	5	6	4	90	–	–	48	37	5	–	1.06	(0.22–4.00)
Sirachainan et al., Thailand 2008 [14]	46	<18	–	33	13	0	161	<18	–	117	44	0	0.90	1.10	(0.50–2.20)
Akar et al., Turkey 1999 [15]	28	<18	–	14	10	4	106	–	–	63	37	6	–	3.9	(0.75–12.10)
Herak et al., Croatia 2009 [16]	59	<16	64.4	20	30	9	112	9.8 ± 4.3	69.9	46	56	10	0.333	1.82	(0.58–5.77)
Komitopoulou et al., Greece 2006 [17]	90	5.5 ± 4.8	–	36	46	8	103	3.9 ± 4.0	–	46	39	18	0.094	1.20	(0.70–2.10)
Cardo et al., Spain 2000 [18]	21	<18	–	6	9	6	28	–	–	7	17	4	ND	ND	ND
Nowak-Göttl et al., Germany 1999 [19]	148	0.5–16	47	113		35	296	0.5–16	47	265		31	<0.0001,	2.60	(1.53–4.50)
Barreirinho et al., Portugal 2003 [20]	21	5.3	–	9	11	1	114	–	–	55	46	13	0.298	0.37	(0.05–2.98)
Prengler et al., UK 2001 [21]	118	0.5–17	–	96		22	78	–	–	69		9	–	1.80	(0.76–4.00)
McColl et al., UK 1999 [22]	37	<15	–	30		7	158	–	–	139		19	–	1.70	(0.60–4.50)
Summary	822					120	1,552					144			

*OR* odds ratio, *CI* confidence interval, *ND* non-detectable due to low number of cases and controls

^a^Data from included publications

A total number of 2,374 individuals were involved in this meta-analysis, including 822 children and adolescents after ischemic stroke and 1,552 control subjects. In all of the 15 studies, different racial populations were analyzed, however Caucasians were the most common.

The association between 677C>T polymorphism within *MTHFR* gene was observed in three of the analyzed studies [8, 9, 19]. The strongest relationship between *MTHFR* polymorphism and childhood ischemic stroke was observed by Biswas et al. [9] although the result concerned synergistic effect between *HPA*-*1* and *MTHFR* polymorphisms. The other of the included studies showed no relations between *MTHFR* polymorphism and ischemic stroke in childhood [10–18, 20–22] (Table 1).

### *MTHFR* polymorphism and childhood stroke

Summary frequencies of *MTHFR* genotypes and alleles from all the studies included in the meta-analysis are presented in Table 1.

There was significant heterogeneity between the analyzed studies observed for the following analyses: I^2^ = 47.4 %, *p* = 0.025 in case of TT versus CC + CT analysis and therefore random effects model with Dersimonian Laird test was used to analyze this combination.

**Fig. 1:**
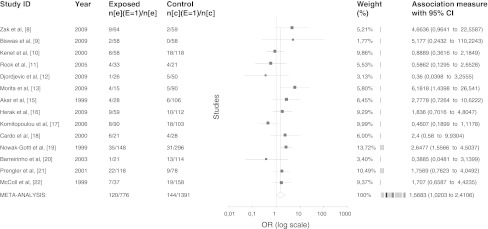
Meta-analysis of association between TT genotype of *MTHFR* gene and pediatric ischemic stroke. OR and 95 % CI of the TT genotype versus CC + CT were calculated from data presented in individual studies. A random effects model with the method of Dersimonian Laird was used to calculate pooled weighted OR. One study was excluded from the calculation because of the lack of TT genotype, both in cases and controls [14]

The pooled analysis showed that TT genotype of *MTHFR* gene is more common in stroke patients than in controls when compared to CC + CT genotypes (*p* = 0.0402, OR = 1.57, 95 % CI 1.02–2.41) (Fig. 1). For the *MTHFR* gene polymorphism, the Egger’s test did not reveal presence of a publication bias (*z* = 0.22, *p* value (two-tailed) = 0.825).

**Fig. 2:**
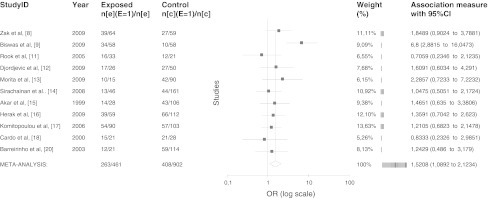
Meta-analysis of association between carriers of T allele (individuals with CT + TT genotypes) of *MTHFR* gene and pediatric ischemic stroke. OR and 95 % CI of the CT + TT genotypes versus CC were calculated from data presented in individual studies. A random effects model with the method of Dersimonian Laird was used to calculate pooled weighted OR. Four of the studies were excluded from the calculation due to lack of accurate data of CT genotype, both in cases and controls [10, 19, 21, 22]

Additionally, in 11 of the studies we calculated association between carriers of *MTHFR* T allele (subjects with CT + TT genotypes) and wild-type homozygous. The pooled analysis showed that carriers of T allele are more common in stroke patients than in controls when compared to individuals with CC genotypes (*p* = 0.014, OR = 1.52, 95 % CI 1.09–2.12) (Fig. 2).

### Sensitivity analysis

According to our calculation, distribution of the *MTHFR* genotypes in controls differed from Hardy–Weinberg equilibrium (HWE) in one of the analyzed studies, what suggested possible genotyping errors or population bias (χ^2^ = 4.033, *p* = 0.045), although the result was close to the bound of significance. Exclusion of this study did not alter the results, thus suggested stability of the present meta-analysis.

## Discussion

In the present study we investigate association between 677C>T polymorphism in *MTHFR* gene and ischemic stroke in children. Data concerning relations between genetic risk factors and pediatric stroke are scarce in comparison to adult stroke analyses. In some pediatric patients the etiology of stroke is not fully understood, however, we may, as it was previously suggested, attribute it to the significant role a genetic factor may play in the development of ischemic stroke [4]. The *MTHFR* 677C>T polymorphism may be related to higher levels of homocysteine and in consequence to a higher stroke risk [6, 7, 30]. According to Fowler [31] the homozygous variant of *MTHFR* gene is present in 5–18 % of the population. Hyperhomocysteinemia may consequently lead to endothelial dysfunction of arteries, an early step in the development of atherosclerosis [7]. Homocysteine also stimulates elevation of superoxide anion, platelet aggregation and decreases nitric oxide bioavailability [32]. Thus elevated homocysteine is an established risk factor in venous and arterial thrombosis although there are some discrepancies on association between *MTHFR* polymorphism and the increased level of HCys. Some studies indicate such relations with Hcys levels elevated over 25 % in TT individuals compared to subjects with CC genotype [6]. It was also demonstrated, in a general population, that individuals with TT genotype and a low plasma folate level or a low folate intake, had higher total plasma homocysteine concentrations than subjects with CT and CC genotypes [33]. On the other hand, Alsayouf et al. [34] found no relation between TT genotype and homocysteine concentration and observed minimal role of 677C>T polymorphism and stroke in children. According to Unal et al. [35] *MTHFR* polymorphism analyzed alone, without level of homocysteine, had no important role in pediatric ischemic stroke. Among analyzed studies, the association between 677C>T polymorphism within *MTHFR* gene was observed in three reports [8, 9, 19], the rest of the studied data did not confirm the relation [10–18, 20–22]. These discrepancies may be mainly due to a low number of patients in the analyzed groups.

Under these controversial results, we decided to perform meta-analysis based on a larger group of cases and controls than in single studies to estimate whether there is any relation between TT homozygous state and pediatric ischemic stroke.

Results obtained from the analyzed group of cases and controls confirmed that TT genotype of 677C>T polymorphism as well as carrier-state of T allele are associated with the development of childhood ischemic stroke. Caucasians were the most common race among the analyzed subjects. Racial-ethnic differences in distribution of the 677C>T polymorphism in *MTHFR* gene have already been described [36, 37]. Klerk et al. [38] demonstrated that the association between *MTHFR* 677C>T polymorphism and coronary heart disease was weaker in European populations, than that observed in Asian populations, which might be partially attributable to the difference in folate intake between the two ethnic groups.

Earlier studies on *MTHFR* polymorphism in different populations yielded equivocal results on associations between *MTHFR* 677C>T polymorphism and stroke, both in adults and children. McColgan and Sharma [39] found no relation between *MTHFR* polymorphism and carotid dissection, a common cause of stroke in young adults accounting for 20 % of strokes. Meta-analysis of three candidate genes: *MTHFR*, *ACE* and *APOE* in Asian population, revealed a significant association of stroke with the *MTHFR* 677C>T polymorphism and *APOE* epsilon 4 alleles, in contrast to *ACE* gene insertion/deletion polymorphism [40]. In another study based on about 7,000 stroke patients, a gradual increase in the ischemic stroke risk with increasing *MTHFR* 677T allele dose was observed, which suggests that *MTHFR* polymorphism may play a significant role in genetic susceptibility to stroke [41]. Meta-analysis of several studies analyzing correlation between *MTHFR* polymorphism and levels of Hcys and stroke suggested that *MTHFR* TT genotype may have a small role in determining susceptibility to ischemic stoke [42]. Kelly et al. [42] have also found correlation between mild-to-moderate hyperhomocysteinemia and stroke in the group of about 2,500 stroke patients. On the other hand Wu and Tsongalis [43] confirmed the association between the TT homozygous variant of *MTHFR* gene and coronary artery disease (CAD) but denied its connections with the stroke.

Previous data concerning the role of *MTHFR* polymorphism and CAD also confirmed the fact that individuals with the TT genotype had a significantly higher risk of CAD, particularly in the setting of low folate status [38]. The results of meta-analysis carried out by Klerk et al. [38] proved that impaired folate metabolism, resulting in high homocysteine levels, is related to an increased risk of CAD. In another group of patients with CAD it was found that carrier-state of T allele of *MTHFR* polymorphism, together with other polymorphic variants of candidate genes increased the risk of the disease, especially in women [44]. Another data of *MTHFR* polymorphism on venous thrombosis demonstrated that *MTHFR* TT genotype was associated with a 20 % higher risk of the venous thrombosis development than in case of to the CC genotype [45].

The present study has some limitations: the largest one is the variability between populations of the prevalence of *MTHFR* TT genotype in controls which ranges from 0 % [9, 14] to 19 % [11]. Such differences may be due to a different number of individuals recruited to the analyzed studies. Previously, Franco et al. [37] showed that 677C>T polymorphism in *MTHFR* gene has a significantly heterogeneous distribution among different ethnic groups and this may explain geographical or racial differences that are linked to the risk of cerebrovascular diseases. Another limitation of our meta-analysis is the fact that in some of the analyzed data included neonates with stroke which also became inclusion criterion in the present meta-analysis, although some publications differentiate neonatal stroke from childhood stroke [46].

To the best of our knowledge, this is the first meta-analysis concerning relationship between *MTHFR* polymorphism and the risk of pediatric stroke, including data from Polish studies. What is more, the present meta-analysis encompasses over eight hundred children after ischemic stroke and provides more reliable evidence on the role of the 677TT genotype in the pathogenesis of pediatric stroke than scarce data based on small group of patients.
